# Combating Antimicrobial Resistance: Innovative Strategies Using Peptides, Nanotechnology, Phages, *Quorum Sensing* Interference, and CRISPR-Cas Systems

**DOI:** 10.3390/ph18081119

**Published:** 2025-07-27

**Authors:** Ana Cristina Jacobowski, Ana Paula Araújo Boleti, Maurício Vicente Cruz, Kristiane Fanti Del Pino Santos, Lucas Rannier Melo de Andrade, Breno Emanuel Farias Frihling, Ludovico Migliolo, Patrícia Maria Guedes Paiva, Paulo Eduardo Teodoro, Larissa Pereira Ribeiro Teodoro, Maria Lígia Rodrigues Macedo

**Affiliations:** 1 Protein Purification and Biological Functions Laboratory, Faculty of Pharmaceutical Sciences, Food and Nutrition, Federal University of Mato Grosso do Sul (UFMS), P.O. Box 549, Campo Grande 79050-010, MS, Brazil; ana.cristina@ufms.br (A.C.J.); apboleti@gmail.com (A.P.A.B.); mauricio.cruz@ifg.edu.br (M.V.C.); kristianefdp@gmail.com (K.F.D.P.S.); rannier.andrade@outlook.com (L.R.M.d.A.); brenoemanuelfarias@gmail.com (B.E.F.F.); 2 Federal Institute of Education, Science and Technology of Goiás, Goiânia 74055-110, GO, Brazil; 3 Postgraduate Program in Biotechnology, Dom Bosco Catholic University, Campo Grande 79117-900, MS, Brazil; rf4900@ucdb.br; 4 Biochemistry Department, Federal University of Pernambuco (UFPE), Recife 50670-901, PE, Brazil; patricia.paiva@ufpe.br; 5 Chapadão do Sul Campus, Federal University of Mato Grosso do Sul (UFMS), Chapadão do Sul 79560-000, MS, Brazil; paulo.teodoro@ufms.br (P.E.T.); larissa_ribeiro@ufms.br (L.P.R.T.)

**Keywords:** antimicrobial peptides, biofilm disruption, combination therapies, drug delivery systems, multidrug resistance

## Abstract

Antimicrobial resistance (AMR) has emerged as one of the most pressing global health challenges of our time. Alarming projections of increasing mortality from resistant infections highlight the urgent need for innovative solutions. While many candidates have shown promise in preliminary studies, they often encounter challenges in terms of efficacy and safety during clinical translation. This review examines cutting-edge approaches to combat AMR, with a focus on engineered antimicrobial peptides, functionalized nanoparticles, and advanced genomic therapies, including Clustered Regularly Interspaced Short Palindromic Repeats-associated proteins (CRISPR-Cas systems) and phage therapy. Recent advancements in these fields are critically analyzed, with a focus on their mechanisms of action, therapeutic potential, and current limitations. Emphasis is given to strategies targeting biofilm disruption and quorum sensing interference, which address key mechanisms of resistance. By synthesizing current knowledge, this work provides researchers with a comprehensive framework for developing next-generation antimicrobials, highlighting the most promising approaches for overcoming AMR through rational drug design and targeted therapies. Ultimately, this review aims to bridge the gap between experimental innovation and clinical application, providing valuable insights for developing effective and resistance-proof antimicrobial agents.

## 1. Introduction

The AMR is one of the greatest public health threats of the 21st century, with profound implications for morbidity and mortality, healthcare system sustainability, and the global economy [1]. The excessive and inappropriate use of antimicrobials in human medicine, veterinary practice, and agriculture has accelerated the selection of multidrug-resistant pathogens like methicillin-resistant *Staphylococcus aureus* (MRSA), carbapenem-resistant *Pseudomonas aeruginosa*, and extended-spectrum β-lactamase (ESBL), limiting treatment options and increasing infection-related costs [2].

Between 1990 and 2021, AMR caused 4.71 million deaths associated with resistance, including 1.14 million direct cases in a global study in 204 countries and territories. During this period, AMR-related mortality in children under 5 significantly declined due to public health advances, while the elderly (70+) faced an over 80% increase, reflecting population aging and heightened vulnerability to hospital-acquired infections [3].

AMR infections are a major global health threat, causing a much higher mortality burden than previously estimated [4]. Projections for 2050 are alarming: AMR may cause 1.91 million direct and 8.22 million associated deaths globally [3]. Mitigation strategies should span from policy-level actions (antimicrobial stewardship, surveillance systems) to technological innovations (rapid diagnostics, novel drug delivery systems) and fundamental research (new antibiotics targeting Gram-negative bacteria).

Thus, AMR is a global crisis demanding urgent action, including investments in surveillance, infection control, novel antibiotics, and antimicrobial stewardship policies to avert millions of deaths in the coming decades [5,6].

## 2. Systemic Impacts and Control of AMR

### 2.1. Public Health Impact

Infections caused by multidrug-resistant (MDR) microorganisms constitute a critical public health challenge worldwide due to their substantial contribution to increased morbidity and mortality. Current evidence indicates that MDR infections are associated with higher fatality rates, prolonged hospital stays, and increased risk of complications, thereby imposing significant burdens on healthcare systems. For instance, carbapenem-resistant bacterial infections, often caused by pathogens such as *Klebsiella pneumoniae* and *Acinetobacter baumannii*, exhibit mortality rates exceeding 50% [5].

In the case of tuberculosis (*Mycobacterium tuberculosis*), multidrug-resistant tuberculosis (MDR-TB) reduces cure rates to approximately 55% compared to 85% for drug-sensitive cases [6]. Additionally, MRSA infections remain a major cause of healthcare-associated morbidity [7].

These findings underscore the urgent need for integrated surveillance, robust infection prevention and control measures, and the development of novel therapeutic strategies to effectively combat MDR pathogens and mitigate their impact on public health.

### 2.2. Economic Impact

The burden of AMR is disproportionately higher in regions with fragile health systems, such as Sub-Saharan Africa [4] and Southeast Asia [6], where the lack of laboratory infrastructure, weak epidemiological surveillance, and limited healthcare regulations hinder effective control of resistant pathogen spread. In these settings, the absence of structured antimicrobial stewardship programs and unregulated access to antibiotics fosters the emergence and persistence of resistance mechanisms.

Moreover, the globalization of trade and human mobility has accelerated the transnational transfer of resistance genes such as mcr-1 (mediator of colistin resistance) and NDM-1 (New Delhi metallo-β-lactamase-1), which have been detected across multiple continents [8]. Genes like NDM-1 now show intercontinental transfer via plasmid conjugation, with identical sequences identified in Indian poultry farms and European intensive therapy units. This underscores the role of international exchange in amplifying AMR as a global public health threat [9]. Such realities reinforce the need for coordinated strategies among nations and multilateral organizations to mitigate the spread of genetic determinants of resistance.

### 2.3. Healthcare System Burden

AMR strains health systems, especially in low- and middle-income countries. Treating resistant infections often requires last-line therapies (colistin, linezolid), which are more toxic and costly. While the U.S. spends approximately USD 1.38 billion annually on AMR-related hospital costs, Sub-Saharan Africa experiences 23% higher AMR-associated mortality rates due to diagnostic limitations [7]. In resource-limited settings, inadequate diagnostics lead to empirical treatment, exacerbating resistance. Investments in precise diagnostics, equitable access to therapies, and strict prescribing policies are urgently needed. The 20-fold cost difference between first-line and last-line antibiotics (ampicillin: While ampicillin costs USD 2 per dose, colistin shows a significantly higher price (USD 40/dose)) cripples low- and middle-income countries’ (LMICs) budgets [10]. These systemic challenges highlight the importance of adopting multi-sectoral strategies, such as the One Health approach, to mitigate the increasing threat of AMR to human, animal, and environmental ecosystems.

### 2.4. Multisectoral Strategies for Effective Antimicrobial Resistance Control

Addressing AMR requires coordinated, multidimensional, and sustained strategies at a global level. The One Health approach endorsed by the WHO, the World Organisation for Animal Health (WOAH), and the Food and Agriculture Organization (FAO) provides a vital integrative framework that unifies human, animal, and environmental health sectors to collectively address the escalating threat of AMR [11].

Within this scope, antimicrobial stewardship programs are particularly noteworthy. Their implementation in hospitals and communities promotes rational and responsible antimicrobial use, improves clinical practices, and reduces selective pressure on microorganisms [9].

Concurrently, therapeutic innovation efforts are essential to expand the available pharmacological arsenal amid the growing inefficacy of conventional antimicrobials. Strategies such as novel antibiotic development, phage therapy, immunomodulation, and antimicrobial peptides (AMPs) have gained increasing attention as promising alternative approaches [12]. Finally, global epidemiological surveillance—exemplified by systems like the WHO-coordinated Global Antimicrobial Resistance and Use Surveillance System (GLASS)—constitutes a cornerstone for the early detection of emerging resistance patterns, thereby informing evidence-based public policies [6].

## 3. Molecular Mechanisms of Bacterial Resistance

AMR is an evolutionary phenomenon resulting from genetic mutations and the horizontal transfer of resistance genes, mediated by mobile genetic elements such as plasmids, transposons, and integrons [13]. Among the pathogens of greatest concern, classified as priority by the WHO [14], are CRKP (carbapenem-resistant *K. pneumoniae*), MRSA, and MDR-TB, which have developed sophisticated resistance mechanisms, including the production of carbapenemases (blaKPC), altered penicillin-binding proteins (mecA), and mutations in RNA polymerase (rpoB), particularly for rifampicin [15]. The global spread of AMR is driven by various factors, such as the indiscriminate use of antibiotics, whether through self-medication or inappropriate prescription [16], the lack of regulation in LMICs, and the scarcity of therapeutic innovations. In recent decades, only two new classes of antibiotics have been approved, reflecting stagnation in antimicrobial development [17].

In this section, we will discuss bacterial resistance to antimicrobials, which is mediated by four main biochemical mechanisms that act synergistically, allowing bacterial survival even under the selective pressure exerted by antibiotics.

### 3.1. Enzymatic Inactivation of Antibiotics

This system represents one of the oldest and most effective mechanisms of bacterial resistance. In this process, bacteria produce specific enzymes capable of chemically modifying or structurally degrading antimicrobial agents, neutralizing their activity [18]. Among the most significant enzymes are β-lactamases, which promote the hydrolysis of the β-lactam ring, a structure essential for the action of β-lactam antibiotics. This group includes both extended-spectrum β-lactamases (ESBLs) and carbapenemases, which are responsible for concerning resistance to penicillins, cephalosporins, and carbapenems in various Gram-negative pathogens [19,20]. ESBLs such as TEM-52 and CTX-M-15 are enzymes that efficiently hydrolyze third-generation cephalosporins, rendering these antibiotics clinically ineffective against the bacteria that produce them. Their catalytic efficiency (k_cat_/K_m_) can reach up to 10^6^ M^−1^ s^−1^, explaining the high level of resistance observed in clinical settings [20].

In addition to β-lactamases, a critical system involves aminoglycoside-modifying enzymes (AMEs), which chemically alter these molecules through acetylation, adenylation, or phosphorylation reactions, drastically reducing their affinity for bacterial targets. The production of these enzymes, often associated with mobile genetic elements such as plasmids and transposons, facilitates the rapid spread of resistance genes among different bacterial species. This exacerbates the current AMR crisis and limits available therapeutic options [21,22,23].

### 3.2. Molecular Target Modification

A fundamental mechanism of bacterial resistance involves structural modifications of antibiotic target sites, preventing drug recognition and binding while preserving the target’s biological function [23]. This strategy, known as target modification, represents one of the most evolutionarily refined resistance mechanisms, as it maintains cellular viability while rendering antibiotics ineffective.

The paradigmatic example occurs in MRSA, where the acquisition of the alternative penicillin-binding protein PBP2a (encoded by the mecA gene) reduces β-lactam affinity by approximately 1000-fold compared to native PBPs [24]. This single modification confers cross-resistance to nearly all β-lactam antibiotics, including penicillins, cephalosporins, and carbapenems. Similarly, in Gram-negative pathogens like *Escherichia coli* and *P. aeruginosa*, sequential mutations in the quinolone-resistance-determining regions (QRDRs) of DNA gyrase (gyrA/B) and topoisomerase IV (parC/E) induce allosteric changes that decrease fluoroquinolone binding affinity by 80–95% while maintaining essential enzymatic activity [25].

This mechanism’s evolutionary success stems from three key factors: (1) it preserves target protein function, imposing minimal fitness costs; (2) modifications often require single nucleotide changes, facilitating rapid emergence; and (3) the altered targets frequently remain refractory to next-generation antibiotics within the same class [26]. For instance, newer cephalosporins designed against MRSA’s PBP2a show only marginal improvements in binding kinetics, while novel fluoroquinolone analogues struggle to overcome gyrase mutations.

The clinical impact is particularly severe because target-modified strains maintain full virulence while resisting treatment. Surveillance data reveal that target-mediated resistance accounts for 38% of MRSA infections and 72% of fluoroquinolone-resistant *E. coli* isolates in hospital settings. Moreover, the horizontal transfer of mutated target genes via mobile genetic elements accelerates dissemination, as seen with the global spread of *gyrA* mutations among *Salmonella enterica* serovars [27,28].

This resistance strategy presents unique challenges for drug development, as it requires either (a) compounds that bypass the modified targets entirely (teixobactin targeting cell wall precursors rather than PBPs) or (b) adjuvant therapies that restore target accessibility (efflux pump inhibitors combined with fluoroquinolones) [29]. Current research focuses on structural biology approaches to design antibiotics that accommodate common target modifications while maintaining potency.

Metallic (Ag or ZnO) and polymeric (chitosan or Polylactic-co-Glycolic Acid) NPs are emerging as promising therapeutic strategies against multidrug-resistant microorganisms, acting through synergistic mechanisms of action. NPs compromise the integrity of the bacterial plasma membrane through electrostatic interactions and pore formation, induce oxidative stress via the generation of reactive oxygen species (ROS), inhibit the formation of extracellular matrix biofilms, and interfere with the processes of genetic material replication and protein synthesis [30]. Potential cytotoxicity in eukaryotic cells, mediated primarily by redox imbalances and the activation of inflammatory pathways, poses a challenge for clinical application. Surface engineering strategies, including functionalization with polyethylene glycol (PEG), the optimization of physicochemical parameters (size between 10 and 50 nm, modulation of surface charge), and the development of controlled-release systems tend to improve the therapeutic index [31]. Approaches with conventional antimicrobials allow for the reduction of effective doses, minimizing adverse effects. Nanotechnology for clinical practice also requires rigorous preclinical investigations, with an emphasis on pharmacokinetic studies, chronic toxicity, and immunogenicity assessment, aiming to ensure both antimicrobial efficacy and biosafety in therapeutic applications [32,33].

### 3.3. Efflux Pump in Antimicrobial Resistance

Efflux pumps are essential components of bacterial resistance, actively expelling antibiotics from cells to reduce intracellular concentrations and compromise treatment efficacy. In Gram-negative bacteria like *P. aeruginosa* and *E. coli*, the RND (Resistance Nodulation Division) family plays a major role in multidrug resistance, while in Gram-positive pathogens such as *S. aureus*, MFS (Major Facilitator Superfamily) pumps mediate resistance to tetracyclines and fluoroquinolones [34]. Additionally, ABC (ATP-Binding Cassette) transporters in *Enterococcus* spp. utilize ATP hydrolysis to extrude antimicrobials, enhancing multidrug resistance [35].

The overexpression of efflux pumps not only diminishes drug effectiveness, but also drives the selection of resistant strains, facilitating cross-resistance to multiple antibiotic classes [36]. This complicates therapeutic strategies, highlighting the critical need for efflux pump inhibitors to restore antibiotic susceptibility.

### 3.4. Biofilm-Mediated Antimicrobial Resistance

Biofilms constitute a formidable resistance mechanism wherein bacterial communities encase themselves in a protective extracellular matrix composed of exopolysaccharides (EPS), proteins, and extracellular DNA [37,38]. This complex architecture creates a dual barrier system: the physical impedance of antibiotic penetration through matrix entrapment and chemical neutralization via enzyme-mediated drug degradation [34,35].

The biofilm microenvironment establishes steep chemical gradients that generate distinct metabolic zones. These conditions promote the formation of dormant bacterial subpopulations exhibiting up to 1000-fold greater antibiotic tolerance than planktonic counterparts [39]. Concurrently, the nutrient-limited stressful environment within biofilms elevates mutation rates (up to 10× baseline) and facilitates horizontal gene transfer through transformation and conjugation [40].

These synergistic mechanisms transform biofilms into evolutionary incubators for resistance development. Clinical data demonstrate that biofilm-associated infections require 10–1000× higher antibiotic concentrations for eradication compared to planktonic infections [30], explaining their role in chronic wound, catheter-related, and prosthetic joint infections. The matrix’s protective effects persist even after antibiotic exposure, enabling rapid repopulation and recurrent infections.

### 3.5. Quorum Sensing (QS) System in Antimicrobial Resistance

The QS system, a cell-density-dependent signaling system, critically influences AMR by regulating bacterial gene expression. Through autoinducer molecules, QS coordinates resistance mechanisms such as biofilm formation, upregulation of efflux pumps, and expression of drug-inactivating enzymes [39,40,41].

Emerging quorum quenching approaches, which disrupt QS signaling, show therapeutic potential by inhibiting biofilm formation and restoring antibiotic susceptibility [32]. However, challenges persist, including limited inhibitor specificity and the risk of bacterial resistance to QS antagonists. Advancing these strategies requires deeper mechanistic insights into QS pathways to develop targeted therapies [39,40].

The interplay of QS-mediated resistance mechanisms highlights the urgent need for innovative antimicrobial designs to combat evolving bacterial threats.

## 4. Innovative Approaches in Antimicrobial Development

### 4.1. Inhibition of Cell Wall Synthesis

One of the greatest global public health challenges is undoubtedly the discovery of new antimicrobial agents that are both effective and capable of circumventing microbial resistance mechanisms [2]. The main driver of AMR has been the indiscriminate use of antimicrobial agents, particularly antibiotics. This practice has favored the development of antibiotic resistance genes, complicating the treatment of bacterial, viral, fungal, and parasitic infections [42,43,44]. In this context, the global scientific community has been actively seeking alternatives aimed at discovering novel antimicrobial compounds.

**Figure 1:**
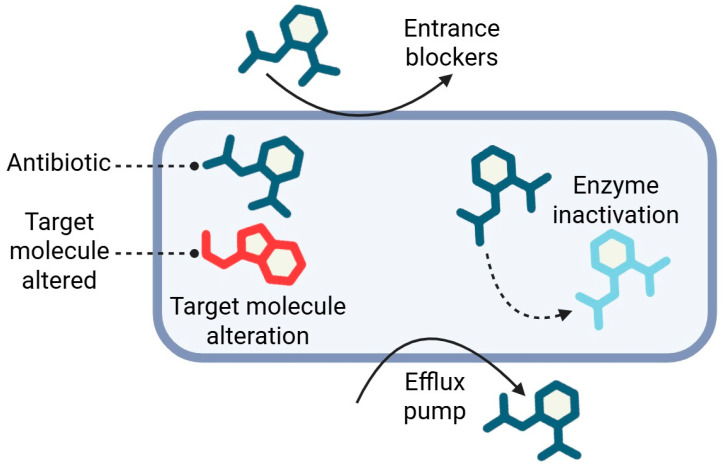
Mechanisms of bacterial antibiotic resistance. Created by Biorender.com.

One promising strategy for antimicrobial development involves inhibiting essential metabolic pathways in microorganisms, which requires a thorough understanding of these pathways. When effectively targeted, such inhibition can compromise cellular viability, leading to microbial death or inactivation. Since metabolic pathways vary among different microbial strains, understanding their specificities is crucial for developing new antimicrobial drugs [45]. This approach, combined with the elucidation of novel molecular targets, enables the development of innovative antimicrobial therapies. In this section, we will describe the key microbial metabolic pathways (Figure 1) being explored for the development of new antimicrobial agents.

#### Inhibition of Peptidoglycan Synthesis

Peptidoglycans are heteropolymers consisting of alternating chains of monosaccharides such as N-acetylglucosamine (GlcNAc) and N-acetylmuramic acid (MurNAc) monosaccharides linked by β1 → 4 glycosidic bonds. The D-lactoyl group of each MurNAc is substituted by a short peptide stem, mostly composed of L-Ala-γ-D-Glu-meso-A2pm (or L-Lys)-D-Ala-D-Ala (where A2pm is 2,6-diaminopimelic acid) in nascent peptidoglycan, with the final D-Ala residue removed in the mature macromolecule [46].

This macromolecule is the primary structural component of bacterial cell walls, providing rigidity, protection against osmotic pressure, and maintenance of cell shape, and serving as an anchoring platform for other cell envelope components, including proteins and polysaccharides. Gram-positive bacteria possess a thick peptidoglycan layer (approximately 40–80 nm), while Gram-negative bacteria have a thinner layer (7–8 nm) associated with an outer membrane [47].

The biosynthesis of peptidoglycan involves complex multi-stage mechanisms (approximately 20 reactions) occurring in three cellular compartments: (1) cytoplasmic synthesis of nucleotide precursors (UDP-GlcNAc to UDP-MurNAc-pentapeptide), (2) membrane-associated synthesis of lipid-linked intermediates, and (3) extracellular polymerization reactions. The peptide stem assembly is catalyzed by Mur ligases (MurC, MurD, MurE, and MurF), which sequentially add L-alanine, D-glutamic acid, meso-diaminopimelic acid (A2pm) or L-lysine, and D-alanyl-D-alanine to UDP-MurNAc [46].

A key regulatory enzyme in this pathway is glucosamine-6-phosphate synthase (GlmS, EC 2.6.1.16), an aminotransferase that irreversibly converts D-fructose-6-phosphate to D-glucosamine-6-phosphate using L-glutamine as a nitrogen source, a critical intermediate for UDP-GlcNAc synthesis [48]. GlmS represents a promising antimicrobial target, as its irreversible inhibition disrupts peptidoglycan precursor production.

Natural (azaserine, albizzin) and synthetic (6-diazo-5-oxo-L-norleucine, N3-fumaroyl-L-2,3-diaminopropionic acid derivatives) glutamine analogues can covalently bind to the N-terminal cysteine residue in GlmS’s glutamine-binding site [49]. Several electrophilic glutamine analogues have been investigated as irreversible GlmS inhibitors, including γ-dimethylsulfonium ((CH_3_)_2_S^+^) derivatives among the most potent reported inhibitors [50].

Natural products like tetaine (bacilysin) from *Bacillus subtilis* exhibit dual antibacterial/antifungal activity [51]. Anticapsin acts as a competitive inhibitor against L-glutamine and is non-competitive against D-fructose-6-phosphate, but irreversibly inactivates GlmS in glutamine’s absence [52]. However, non-selective analogues like 6-diazo-5-oxo-L-norleucine or azaserine show limited therapeutic potential due to off-target effects [49].

### 4.2. Inhibition of the Synthesis of Essential Metabolites

#### 4.2.1. Inhibition of Folic Acid Synthesis

Folic acid and its derivatives are essential for bacterial development as they serve as substrates for a series of enzymatic biotransformations. They are involved in the synthesis of vital biomolecules such as amino acids, purines, and pyrimidine nitrogenous bases, as well as the production of transfer RNAs, which are crucial for protein synthesis [53,54,55].

Since bacteria have limited capacity to absorb exogenous folic acid, they must synthesize it de novo. Consequently, inhibiting this pathway represents an effective strategy for developing antimicrobial agents with minimal risk to humans as they lack this metabolic pathway [56].

**Figure 2:**
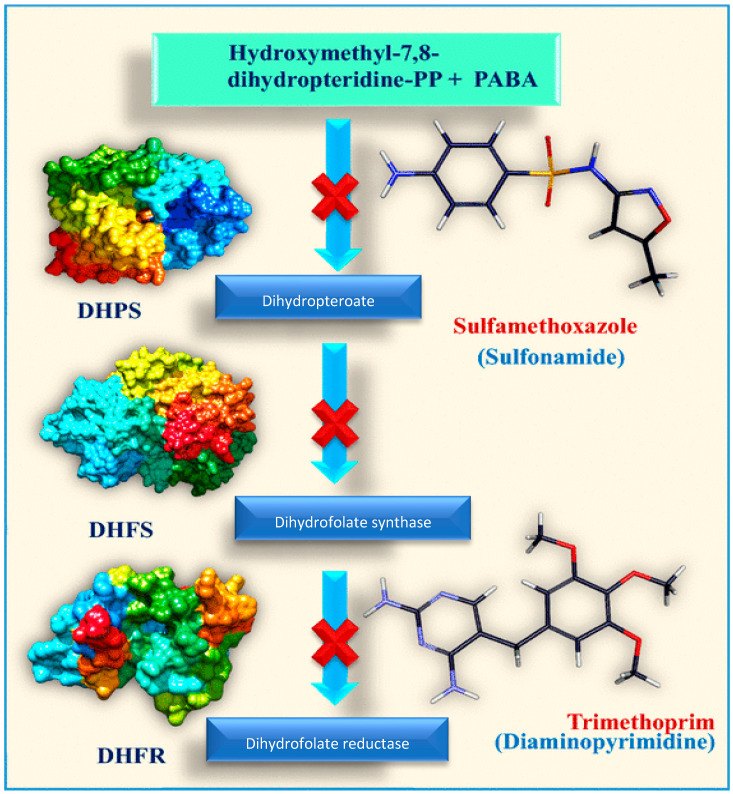
Metabolic pathway of folate synthesis in bacteria, highlighting the target enzymes of inhibitors DHPS (dihydropteroate synthase), DHFS (dihydrofolate synthase), and DHFR (dihydrofolate reductase). Adapted by Pradhan and Sinha [59]. Created in BioRender. Souto, E. (2025) https://BioRender.com/a81d0mj (accessed on 19 July 2025).

Key enzymes in the de novo folate synthesis pathway in bacteria include dihydropteroate synthase (DHPS), dihydrofolate reductase (DHFR), and dihydrofolate synthase (DHFS). Inhibitors targeting these enzymes such as sulfamethoxazole (a DHPS inhibitor) and trimethoprim (a DHFR inhibitor) can disrupt this pathway, ultimately leading to cell death [57] (Figure 2).

Trimethoprim [2,4-diamino-5-(3′,4′,5′-trimethoxybenzyl) pyrimidine] is a synthetic therapeutic compound used to treat mild symptomatic urinary tract infections, either alone or in combination with sulfamethoxazole, a dihydropteroate synthase inhibitor that mimics its substrate, p-aminobenzoic acid (PABA). The combination with sulfamethoxazole is believed to enhance therapeutic efficacy, develop bactericidal activity, and reduce microbial resistance rates to individual components [58].

Trimethoprim’s mechanism of action involves blocking the production of the active form of folic acid (tetrahydrofolate) in susceptible organs, compromising endogenous folate synthesis. Since bacterial cell walls are impermeable to folate, this inhibition severely impairs bacterial growth and proliferation. Specifically, trimethoprim competitively inhibits dihydrofolate reductase, which catalyzes the reduction of dihydrofolate to tetrahydrofolate [60]. Despite being the primary antifolate drugs used in recent decades, bacterial resistance to trimethoprim and sulfamethoxazole has increased by over 50%, highlighting the need for new drugs in this antimicrobial approach [61].

Other antifolate drugs include 4-aminosalicylic acid (salicylate class), a prodrug that inhibits dihydrofolate reductase and is commonly used to treat tuberculosis; dapsone (sulfone class), used to treat leprosy; and mafenide (sulfonamide class), used to treat burns to prevent the proliferation of bacteria and fungi. Both dapsone and mafenide act by inhibiting dihydropteroate synthase [62]. In addition to inhibiting the proliferation of bacterial, fungal, and parasitic infections, antifolates have been shown to be effective in inhibiting the growth of malignant cells in proliferating mammals. Thus, drugs that target the inhibition of the folate pathway have substantial impacts on anticancer therapies [63].

#### 4.2.2. Inhibition of Fatty Acid Biosynthesis

Fatty acid synthesis (FAS) occurs through two molecular systems: Type I FAS (FASI) found in mammals and fungi, which involves multifunctional polypeptides with multiple active sites, and Type II FAS (FASII) present in bacteria and plants, consisting of discrete enzymes encoded by individual genes that collectively catalyze all pathway reactions [64]. Bacterial fatty acids play vital physiological roles and are essential not only for cell membrane formation, but also for producing vitamin precursors. This makes FASII inhibition a promising strategy for novel antibiotic development [64,65].

The structural differences between conserved enzymes and the presence of isozymes that catalyze identical chemical reactions make bacterial FASII a narrow-spectrum target, which provides an advantage over traditional broad-spectrum targets. Numerous inhibitors of lipid synthesis have been reported in the literature (Table 1). Additionally, FASII inhibitors typically act on single targets, unlike conventional broad-spectrum antibiotics with multiple targets [66]. The acyl chains of glycerophospholipids, the most abundant and essential bacterial membrane components, are produced via FASII through cyclic 2-carbon elongation steps from acetyl-CoA precursors, mediated by discrete and multifunctional enzymes [67].

Key targets include enoyl-ACP reductases (FabI and its isoforms FabL, FabK, FabV, and InhA), multifunctional enzymes present across microorganisms (bacteria and protozoa) that represent the sole FASII enzyme responsible for bulk lipid and fatty acid biosynthesis. Their inhibition can yield broad-spectrum antibacterial activity [72,79]. Triclosan, a broad-spectrum antibiotic, blocks lipid biosynthesis by inhibiting enoyl-ACP reductase, though mutations or overexpression of the encoding gene in *E. coli* can confer resistance [80].

### 4.3. Inhibition of Bacterial Respiration

The respiratory pathway is a crucial process for the cellular survival of prokaryotes and eukaryotes that occurs in mitochondria, dynamic organelles that have a double membrane and participate in several cellular processes such as energy production by the tricarboxylic acid (TCA) cycle, oxidative phosphorylation by the electron transport chain, programmed cell death, calcium homeostasis, biosynthesis of other macromolecules, and other cellular signaling pathways [81].

The strategy of inhibiting cellular respiration as a therapeutic target began with studies carried out by Andries and collaborators [82], who evaluated bedaquiline, a diarylquinoline capable of inhibiting *M. tuberculosis*, a drug-resistant microorganism. Studies with mutant strains suggested that the drug targets the proton pump of ATP synthase (F1Fo). The drug was approved by the FDA in 2012 and validated oxidative phosphorylation as a target for the development of anti-tuberculosis drugs [83].

The airway enzyme complex most vulnerable to chemical inhibition for *M. tuberculosis* is cytochrome bcc-aa3 terminal oxidase (QcrB) [84,85]. Independent screens have highlighted a large family of QcrB inhibitors, which are small molecules with high chemical diversity [82,83]. Telacebec (Q203) is considered one of the most potent QcrB inhibitors and the third modern class of anti-tuberculosis drugs with proven potential in humans [86,87].

The nitroimidazole delamanid, another drug used to treat tuberculosis, inhibits both the synthesis of cell wall components and respiration through the release of nitric oxide [88]. Conradie and collaborators [89] demonstrated that pretomanid, another nitroimidazole, can be administered in fixed-dose combination with bedaquiline and linezolid with 90% efficacy against tuberculosis. Other widely known ATP synthase inhibitors are dicyclohexylcarbodiimide, oligomycin, and venturicidin [90]. These inhibitors block ATP synthase activity by interacting with the c subunit or the a/c subunit interface, preventing both mitochondrial and bacterial ATP production [91].

## 5. Emerging Technologies and Novel Targets

Recent scientific advances have significantly expanded the frontiers of antimicrobial discovery through multiple innovative approaches. The investigation of “biological dark matter” was previously unexplored in microbial metabolites with potential antimicrobial properties and has revealed novel chemical scaffolds [92]. Concurrently, breakthroughs in combinatorial chemistry techniques now enable the rapid generation of diverse molecular structures, while comprehensive purchasable small-molecule libraries provide unprecedented access to chemical diversity [93]. These cutting-edge methodologies collectively enhance screening capabilities within chemical space, dramatically increasing the pool of potential drug candidates [94].

However, the discovery of new antibiotics faces significant challenges beyond target identification. While modern approaches can identify and inhibit vital molecular targets in microbial metabolism, this alone does not guarantee therapeutic efficacy. Key obstacles include poor compound penetration through microbial cell envelopes and active efflux mechanisms that reduce intracellular drug concentrations [95].

Recent breakthroughs in structural biology and genomics are helping overcome these barriers. A prime example is darobactin, which targets BamA, an essential outer membrane protein in Gram-negative bacteria [96]. This innovative approach bypasses traditional penetration challenges by interacting with extracellular components of the bacterial cell envelope, demonstrating how novel target selection can address longstanding delivery limitations.

The growing threat of AMR demands innovative therapeutic strategies beyond conventional antibiotics. Phage therapy has re-emerged as a promising alternative, utilizing bacteriophages to specifically target resistant bacterial strains with minimal impact on commensal flora [97]. Similarly, AMPs (natural and synthetic) demonstrate broad-spectrum activity against multidrug-resistant pathogens while reducing the likelihood of resistance development [98]. These approaches address critical gaps in current treatment options.

CRISPR-based antimicrobials represent another paradigm shift in AMR management. By employing targeted gene-editing technology, these systems can selectively eliminate antibiotic resistance genes or directly kill resistant pathogens [84]. Recent studies highlight their potential against biofilm-associated infections and persister cells, which are particularly challenging to treat with conventional antibiotics [99].

Nanotechnology applications offer multifaceted solutions to AMR challenges. Engineered nanoparticles (NPs) can enhance drug delivery, overcome resistance mechanisms, and exhibit intrinsic antimicrobial properties [100]. Polymeric and metallic NPs, lipid-based carriers, and quantum dots have shown promise against bacterial and fungal pathogens [101,102,103].

These emerging technologies (phage therapy, AMPs, CRISPR systems, and nanotechnology) complement traditional discovery methods while addressing key limitations of conventional antibiotics. As discussed in subsequent sections, each approach presents unique advantages and challenges in clinical translation. Their integration into antimicrobial development pipelines offers a comprehensive strategy to combat the escalating AMR crisis [104].

## 6. Biotechnological Approaches and Advanced Therapies in Combating AMR

Bacterial infections associated with biofilm formation represent one of the greatest challenges in contemporary medicine, particularly in the context of AMR. Biofilms provide bacteria with highly effective physical and metabolic protection, hindering antimicrobial penetration and immune system action, while also promoting the persistence of bacterial subpopulations in a metabolically dormant state capable of resisting conventional treatments [37].

**Figure 3:**
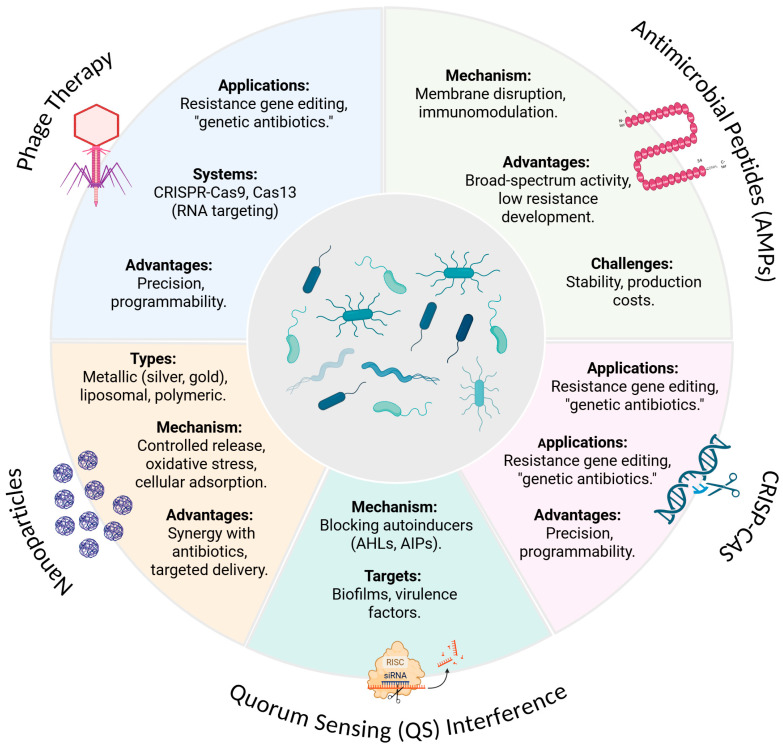
Recent technology strategies against multidrug-resistant bacteria. Academic Individual License granted to UCD/E. B. Souto OBRSS Research Support Scheme (AIUCD241022-cd0d). Created with BioRender.com.

Biofilms contribute to therapeutic failure and chronic infections by limiting antimicrobial diffusion, inducing physiological heterogeneity, and sheltering persister cells. Consequently, biofilms have been recognized as critical new therapeutic targets in combating bacterial resistance [105]. The rise of antibiotic resistance underscores the urgent need for innovative antimicrobial strategies. Nanotechnology, synthetic/bioinspired AMPs, CRISPR-Cas systems, and QS inhibitors represent groundbreaking approaches to overcome these challenges (Figure 3).

### 6.1. Bioinspired and Modified Antimicrobial Peptides

The escalating global spread of multidrug-resistant bacteria represents a critical public health challenge, driving urgent demand for novel therapeutic alternatives [106]. AMPs have emerged as promising candidates due to their broad-spectrum activity against bacteria, fungi, viruses, and parasites, coupled with mechanisms distinct from conventional antibiotics [107]. As fundamental components of innate immunity across species, AMPs primarily act through electrostatic interactions with negatively charged microbial membranes, inducing pore formation, cell lysis, or disruption of essential intracellular processes [108]. However, clinical translation faces hurdles including proteolytic degradation, host cell toxicity, and manufacturing costs, necessitating innovative bioengineering solutions.

Bioinspired design strategies harness nature’s blueprints, derived from organisms reliant on innate immunity (plants, amphibians, fish, and insects), to develop optimized AMPs. By preserving critical structural features (positive charge, amphipathicity, and membrane selectivity) while enhancing therapeutic potential, this approach moves beyond simple replication. Instead, it refines the structural determinants of antimicrobial efficacy for clinical translation. Recent advances integrate machine learning and bioinformatics to accelerate data-driven design, leveraging natural peptide databases [109].

To address the inherent limitations of natural AMPs, researchers have employed advanced structural engineering strategies. A prominent approach involves amino acid substitution (particularly the incorporation of D-amino acids), which markedly improves resistance to proteolytic degradation without compromising antimicrobial activity [110].

Another effective approach is peptide cyclization through disulfide bridges or peptide bonds, which improves structural stability and enzymatic resistance by reducing conformational flexibility. Additionally, hybrid peptide design that combines functional domains from multiple AMPs has proven successful creating molecules with broadened antimicrobial spectra and modulated specificity. Finally, nanocarrier conjugation strategies have emerged as powerful tools to enhance AMP bioavailability and targeted delivery while simultaneously reducing systemic side effects. These engineering approaches collectively address the major pharmacological challenges of native AMPs, enabling the development of next-generation antimicrobial agents with improved therapeutic potential [111,112,113].

Significant achievements consist of modified LL-37 versions exhibiting improved resistance to proteases produced by *P. aeruginosa* and peptides influenced by piscidin that are fine-tuned for heightened antibacterial efficacy while minimizing toxicity to human cells. These developments illustrate the potential of intentional design to connect innate effectiveness with drug development needs [114,115].

Although bioengineered AMPs offer a revolutionary method for addressing AMR, additional progress in formulation, pharmacokinetics, and prolonged safety assessment is necessary for clinical application. The combination of computational design, structural biology, and delivery methods positions AMPs as a cutting-edge solution for tackling emerging microbial challenges.

### 6.2. Nanotechnology Applied to Antibiotics

Nanotechnology is a branch of science and technology that manipulates the properties of matter at the nanoscale, generally from 1 to 100 nm, using properties and phenomena that occur at the nanoscale to develop innovative applications in several areas such as medicine, electronics, energy, and materials science [116]. Recent advances in nanotechnology, especially in the development of NPs for controlled drug delivery, have positively impacted medicine and healthcare. NPs have been shown to be efficient carriers for the controlled release of antibiotics in local drug therapies and infection prophylaxis [116,117].

Nanoparticle-based controlled drug delivery systems offer several key advantages: (1) they improve drug solubility while helping evade immune detection, (2) allow precise control over drug release profiles to target specific sites, and (3) enable simultaneous delivery of multiple therapeutic agents [115,118]. Thus, these systems can improve the pharmacokinetic profile and therapeutic index of the drug when compared to systems involving free drugs, which is why several formulations of antimicrobial NPs have been developed [119,120,121,122] from inorganic materials, liposomes, polymers, dendrimers, and others.

Several studies have demonstrated that the combination of metallic nanoparticles with antimicrobial agents can significantly enhance the activity against resistant microorganisms. Gold nanoparticles associated with antimicrobial peptides were able to reduce the activity of resistant isolates by more than 85% [123]. Similarly, amoxicillin-loaded silver nanoparticles exhibited inhibitory effects against *S. aureus*, *E. coli*, and *Candida albicans*, with reductions in minimum inhibitory concentrations (MICs) and inhibition rates exceeding 90% [124]. Moreover, silver nanoparticles combined with ampicillin, oxacillin, and penicillin resulted in inhibition zones larger than 20 mm [125].

Selenium nanoparticles associated with levofloxacin enhanced the inhibition of MRSA by 70% [126]. Additionally, silver nanowires combined with norfloxacin, and streptomycin reduced the required antibiotic doses against resistant *E. coli* by up to 90% [127]. Furthermore, the association of silver nanoparticles with various antibiotics led to an increase of up to 147% in efficacy against resistant strains of *S. aureus* and *P. aeruginosa*, along with a 54% reduction in the viability of *S. aureus* harboring the *mecA* gene, highlighting the potential of these nanosystems in overcoming bacterial resistance [128,129]. Some key studies that developed NPs with antimicrobial evaluation between 2020 and 2025 are represented in Table 2.

In many treatments involving bacterial infections, antibiotics are administered by systemic routes reaching widely distributed pathogenic bacteria. Alternatively, antibiotics can be administered at the site of infection to maximize drug concentration in a critical region and, as a benefit, there is a potential reduction in the development of resistance caused by systemic exposure [132,133]. In this context, controlled delivery systems of NPs present themselves as an alternative for the treatment of local infections.

Taheri and collaborators [129] developed solid lipid nanoparticles (SLNs) containing ciprofloxacin. This study evaluated the efficacy of ciprofloxacin-loaded SLNs (Cip-SLN) against *P. aeruginosa* and ampicillin–vancomycin-loaded SLNs (Amp-Van-SLN) against *S. aureus* in wound infections. Antibiotics encapsulated in SLNs via double emulsion demonstrated sustained antibacterial effects, with nanoparticle formulations showing superior inhibition after 72 h compared to free drugs, despite initial lower activity at 24 h. In vitro assays (well diffusion, MIC) and murine wound models confirmed enhanced antibacterial activity and improved healing (evidenced by increased epithelial thickness) in SLN-treated groups versus controls. SLNs facilitated targeted antibiotic accumulation at infection sites with slow-release kinetics, reducing bacterial resistance risks while maintaining biocompatibility (no cytotoxicity at ≤400 µg/mL).

The article by Gafar and collaborators [130] discusses the innovative use of peptides in nanosystems to improve the diagnosis and treatment of bacterial sepsis. The authors highlight how peptide-functionalized nanoparticles can detect pathogens early and deliver therapeutic agents in a targeted manner, reducing bacterial resistance and side effects. This approach combines antimicrobial and anti-inflammatory peptides into nanotechnological platforms, offering promising solutions for managing this critical condition, despite challenges in clinical translation.

Inorganic nanomaterials, such as metal and metal oxide NPs, are widely explored in antimicrobial approaches due to their advantages such as low cost, long duration, reduced toxicity, and intrinsic antimicrobial activity [131,132,134]. Caciandone and collaborators [135] functionalized magnetite NPs with streptomycin and neomycin, obtaining a stable nanomaterial, and in cell viability and antimicrobial tests, they demonstrated biocompatibility in human diploid cells and an antibacterial effect against Gram-negative (*P. aeruginosa*) and Gram-positive (*S. aureus*) opportunistic bacteria.

Magnetite NPs conjugated with vancomycin exhibited antibacterial activity against Gram-positive (*B. subtilis* and *Streptococcus*) and Gram-negative (*E. coli*) bacteria. Furthermore, the NPs conjugated with vancomycin were effectively bound to the bacterial cell wall, promoting bacterial separation and growth inhibition [136]. Another class of materials suitable to produce NPs as antimicrobial drug carriers are natural or synthetic polymers, materials that exhibit high versatility, have diverse chemical compositions, and are considerably amenable to functionalization [137,138].

Some synthetic polymers used as nanocarriers include poly(lactic acid) (PLA), PEG, and polycaprolactone (PCL) [139,140,141]. Natural polymers include chitosan, alginates, gelatin, and gliadin [142]. Polymeric NP systems as carriers for antibiotic combinations have been reported. Poly(d-L-lactide-co-glycolide) NPs containing a combination of rifampicin and azithromycin were shown to be more effective in in vitro tests when compared with the free drugs against persistent chlamydial infection [143]. Clarithromycin and omeprazole were encapsulated in gliadin NPs and exhibited in vivo efficacy against *Helicobacter pylori* bacteria [144].

A study conducted by Sattari-Maraji [123] proposed the synthesis of gold NPs conjugated with a cationic hydrophilic peptide (Jellein-I-Cys-GNPs). For this purpose, a peptide sequence of Jellein-I was synthesized with the addition of a cysteine amino acid at its C-terminus (Jellein-I-Cys) to establish a covalent bond and ensure a higher peptide concentration on the surface of the developed NPs. In the study, the authors evaluated the antimicrobial activity of Jellein-I-Cys-GNPs against *MRSA ATCC 43300* and a clinical MRSA isolate. The results showed that free Jellein-I-Cys (unconjugated peptide) and Jellein-I-Cys-GNPs (conjugated form) exhibited MIC (minimum inhibitory concentration) and MBC (minimum bactericidal concentration) values of 512 μM and 94 μM, respectively. According to the findings, the authors demonstrated that Jellein-I-Cys conjugated with GNPs exhibited enhanced antibacterial activity, likely due to the increased local concentration of peptides around the GNPs. This, in turn, enhanced the positive charge density, making them more readily available to interact with the negatively charged bacterial membrane. Jellein-I-Cys-GNPs demonstrated potent antibiofilm activity against MRSA, killing 27.4–81.8% of biofilm cells and reducing metabolic activity by 77.8–88.8% at 188–376 μM. Unlike vancomycin (requiring 64× MIC), they disrupted pre-existing biofilms at low concentrations, causing cell death, leakage, and structural damage, as confirmed by SEM. This highlights their potential as an effective alternative for combating resistant MRSA biofilms.

### 6.3. CRISPR-CAS as an Antimicrobial Strategy

The CRISPR-Cas system (Clustered Regularly Interspaced Short Palindromic Repeats (CRISPR)-associated proteins) originally emerged as a bacterial defense mechanism against viruses (bacteriophages). First discovered in *E. coli* in the 1980s, its function as an “adaptive immune system” in bacteria was only elucidated in the 2000s [145]. Since 2012, with the development of the CRISPR-Cas9 technique for gene editing, this tool has changed biotechnology, allowing precise modifications to DNA [146].

Recently, CRISPR-Cas has been explored as an innovative antimicrobial strategy aimed at combating pathogenic bacteria, especially multidrug-resistant strains [147].

Unlike traditional antibiotics, which act in a broad and non-specific way, CRISPR-Cas offers an alternative in the fight against pathogens and can be programmed to selectively eliminate resistant bacteria, reversing resistance mechanisms or preventing the formation of biofilms. One example is the use of CRISPR-Cas technology against MRSA, where CRISPR-Cas9 was employed to specifically target the mecA gene, responsible for resistance to β-lactams [148]. This intervention eliminated resistant bacteria and restored sensitivity to oxacillin, demonstrating the potential for re-sensitization to conventional antibiotics.

The strategy known as “phagocentric” therapy represents another advance, where bacteriophages are genetically modified to carry CRISPR-Cas systems, becoming intelligent vectors capable of selectively eliminating pathogens such as *E. coli* and *P. aeruginosa* while preserving beneficial microbiota [149]. This approach was first demonstrated in 2012 and has since been improved to increase its specificity and effectiveness [150].

CRISPR-Cas shows potential for reversing resistance mechanisms. The system’s versatility allows it to act on both DNA and RNA. The CRISPR-Cas13a variant, which acts on RNA, was used to silence β-lactamase enzymes in *K. pneumoniae,* neutralizing their ability to degrade penicillin. CRISPR-Cas13a and related CRISPR systems are being explored for both the detection and control of β-lactamase-producing *K. pneumoniae* [151,152,153]. Another application was with the selective elimination of vancomycin resistance plasmids in *E. faecalis*, an approach that could be extended to other resistance mechanisms mediated by mobile genetic elements.

**Figure 4:**
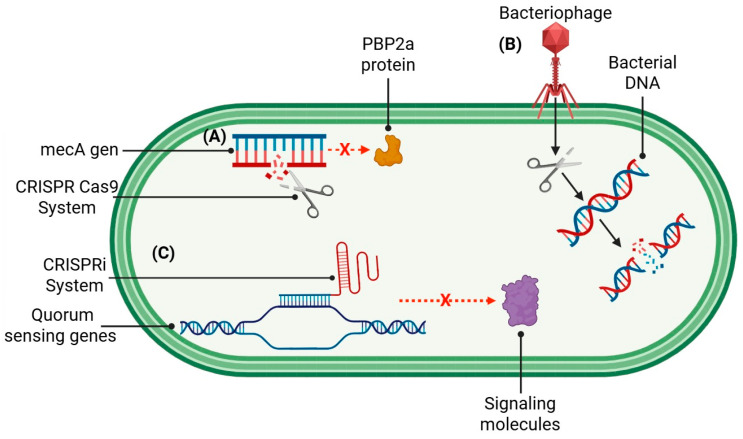
CRISPR-Cas antimicrobial strategies: (**A**) Gene editing in MRSA: CRISPR-Cas9 targets the *mecA* gene (encoding PBP2a), restoring β-lactam susceptibility. (**B**) Phage delivery: Engineered bacteriophages deliver CRISPR-Cas to selectively eliminate pathogens (e.g., *E. coli*, *P. aeruginosa*) while preserving microbiota. (**C**) Biofilm disruption: CRISPRi silences quorum sensing genes in *P. aeruginosa*, inhibiting biofilm matrix formation. Created in BioRender. Souto, E. (2025) https://BioRender.com/a2dd7iy (accessed on 19 July 2025).

The fight against bacterial biofilms benefits from this technology. An approach has been developed using CRISPRi (CRISPR interference) to silence quorum sensing genes in *P. aeruginosa*, significantly reducing the formation of the protective extracellular matrix that characterizes biofilms [154]. This strategy has proven effective in the context of chronic wounds, where biofilms are frequent and difficult to eradicate. The general schematic of the CRISPR-Cas system is shown in Figure 4.

The recent advances include the development of “programmable antibiotics”, which combine the precision of CRISPR-Cas with innovative delivery systems. Lipid NPs loaded with CRISPR-Cas9 have demonstrated efficacy in models of pulmonary infection by *M. tuberculosis* [53], while genetically modified probiotic bacteria, such as *Lactobacillus*, have been used as vectors to deliver CRISPR systems against *Clostridioides difficile* in the intestinal environment [155].

Despite the enormous potential, the field still faces challenges, where efficient delivery of CRISPR components to infected tissues, the risk of off-target editing, and possible immune responses against the system’s components are obstacles that need to be overcome. However, the development of new variants such as Cas13 (which acts on RNA) and the evolution of nanotechnological delivery systems are paving the way for increasingly effective solutions [156]. Against this backdrop of growing AMR, CRISPR-Cas is emerging as one of the most promising tools for a new era of precision antimicrobial therapies that is capable of transforming infection-fighting studies.

### 6.4. Quorum Sensing Inhibition as a Strategy for Antibacterial Activity

Molecular communication is an emerging research field that explores chemical signaling systems in which information is encoded, transmitted, and decoded via the emission of signaling molecules [157]. This nanoscale biological phenomenon involves transmitter nanomachines releasing specific signaling molecules—autoinducers (AIs)—into the environment, which then diffuse to receiver nanomachines that decode the chemical signals [158].

QS is a bacterial communication mechanism that enables cells to share information about population density and coordinate behavioral changes when reaching a critical threshold. This system regulates various processes including virulence factor expression, sporulation, biofilm formation, drug resistance, conjugation, and motility [157,158].

AMR, a global health crisis, stems partly from bacterial QS mechanisms that counteract antibiotic effects. These QS-mediated resistance mechanisms include both the secretion of antibiotic-degrading enzymes (β-lactamases) and the formation of protective biofilms [159,160]. QS inhibition strategies aim to disrupt bacterial coordination, preventing virulence gene expression and biofilm formation that complicate antibiotic treatment [161]. Potential QS interference approaches include inhibition of AI synthesis, AI receptor antagonism, blockade of receptor-binding targets, AI sequestration, AI degradation using catalytic antibodies (abzymes) or enzymes (lactonases), inhibition of AI secretion/transport, and antibody-mediated receptor blockade [162].

Acyl-homoserine lactones (AHLs), essential QS components, are constitutively produced by bacterial cells [163,164]. Increasing bacterial density elevates AHL levels, which, upon interaction with LuxR-family transcription factors, triggers the expression of specific genes, including those encoding AHL-degrading enzymes [165].

**Figure 5:**
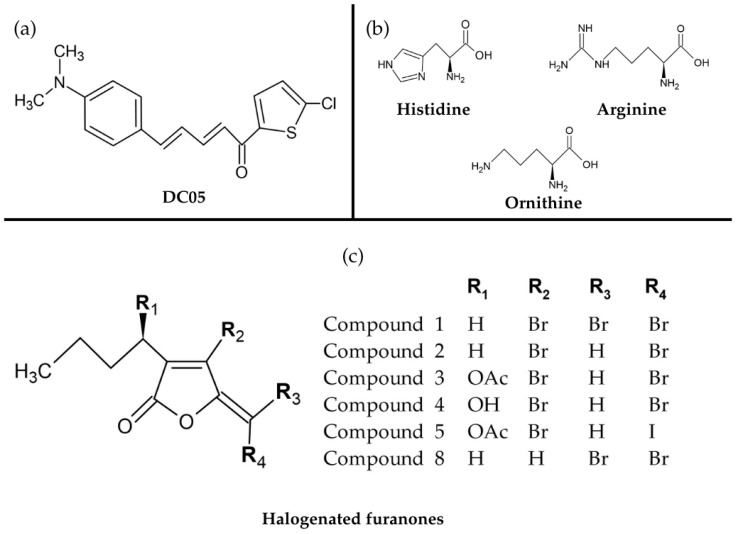
Chemical structures of compounds with anti-quorum sensing activity. (**a**) Chalcone derivative 1-(5-chlorothiophen-2-yl)-5-[4-(dimethylamino)phenyl]prop-2-en-1-one (designated as DC05). Adapted from Nayak and collaborators [161]. (**b**) Selected cationic amino acids with anti-quorum sensing activity against *P. aeruginosa* and *C. violaceum*. Adapted from Abinaya and collaborators [167]. (**c**) Structural variations of five naturally occurring halogenated furanones (compounds **1**–**5**) from the marine macroalga *Delisea pulchra* and a synthetic derivative (compound **8**). Adapted from Manefield and collaborators [169]. All chemical structures were drawn using ACD/ChemSketch (Freeware).

Nayak et al. [161] evaluated the anti-QS activity of chalcone derivative DC05 (Figure 5a), demonstrating antibacterial and antibiofilm effects against *Salmonella typhi* through reduced exopolysaccharide synthesis and downregulation of key QS genes (luxS, lsrB, rpoS, sdiA). Abinaya et al. [166] tested cationic amino acids (Figure 5b) against *P. aeruginosa* and *Chromobacterium violaceum*. Histidine (at MIC/sub-MIC concentrations) showed superior inhibition of virulence factors (elastase, protease, pyocyanin, rhamnolipid, pyoverdine) in *P. aeruginosa* and violacein/chitinase production in *C. violaceum* compared to arginine and ornithine.

**Figure 6:**
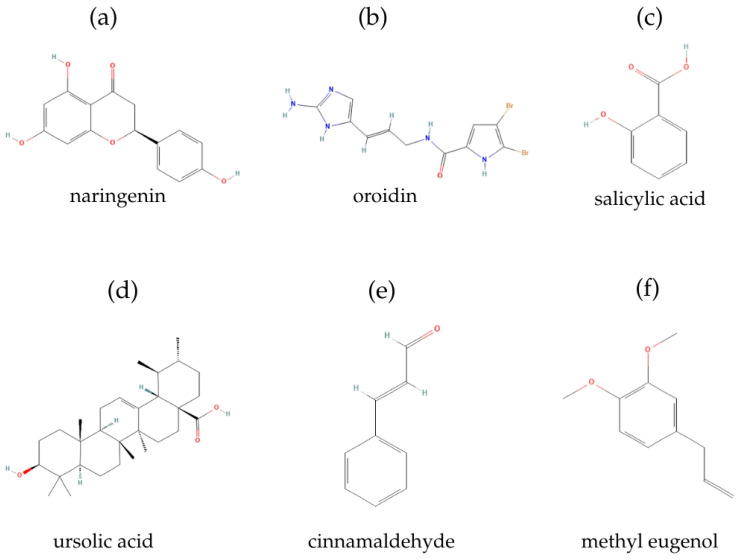
Chemical structures of some phytochemicals with demonstrated anti-QS activity: (**a**) naringenin; (**b**) oroidin; (**c**) salicylic acid; (**d**) ursolic acid; (**e**) cinnamaldehyde; and (**f**) methyl eugenol. Source: PubChem (https://pubchem.ncbi.nlm.nih.gov/, accessed on 25 June 2025).

Numerous plant-derived compounds have been identified as effective QS inhibitors, primarily consisting of secondary metabolites such as phenols and their oxygen-substituted derivatives. A particularly notable example includes halogenated furanones (Figure 5c) produced by the benthic marine alga *Delisea pulchra*. These compounds disrupt QS-regulated behaviors by competitively binding to LuxR-type proteins, effectively preventing biofilm formation [167]. Additional phytochemicals with demonstrated anti-QS activity include naringenin, oroidin, salicylic acid, ursolic acid, cinnamaldehyde, and methyl eugenol (Figure 6), along with various garlic extracts and edible fruit compounds, all exhibiting significant antibiofilm effects against multiple pathogenic species [168].

Recent work by Gonzales et al. [170] has explored the potential of SsoPox lactonase as a broad-spectrum QS disruptor across human, plant, and fish pathogens. Their findings demonstrate that lactonase treatment effectively modifies key pathogenic phenotypes including biofilm formation, motility, and infectivity. In plant pathogens, treatment resulted in reduced production of plant-cell-wall-degrading enzymes in *Pectobacterium carotovorum* and decreased onion maceration caused by *Burkholderia glumae.* For human pathogens, significant inhibition of biofilm formation was observed in *A. baumannii*, *Burkholderia cepacia,* and *P. aeruginosa*. In fish pathogens, the treatment not only inhibited biofilm formation and bioluminescence in *Vibrio harveyi,* but also effectively disrupted QS regulation in *Aeromonas salmonicida*.

Current studies emphasize the critical need for a deeper mechanistic understanding of how plant extracts and phenolic compounds inhibit QS systems [171]. Such fundamental knowledge will enable both the development of novel molecules and structural optimization of existing QS inhibitors to enhance their efficacy. This approach presents promising new avenues for developing innovative antimicrobial therapies that could circumvent conventional resistance mechanisms [172,173]. The combination of phytochemical discovery and enzymatic disruption strategies offers a multifaceted approach to combat AMR at its root by targeting bacterial communication systems.

Bacteriophages, commonly called phages, are viruses that specifically infect bacteria and represent the most abundant biological entities on Earth. These obligate intracellular parasites consist of nucleic acid enclosed within a three-dimensional capsid structure, which may or may not be surrounded by a lipoprotein envelope [174]. Some variants possess a tail with a helical sheath, often equipped with fibers containing receptor-binding proteins that target specific membrane components of host bacteria. While most phages contain double-stranded DNA (dsDNA) genomes, variants with double-stranded RNA (dsRNA), single-stranded DNA (ssDNA), or single-stranded RNA (ssRNA) also exist [175].

Phages are classified into two main types based on their replication cycles: lytic (virulent) and lysogenic (temperate) phages. The lytic cycle involves the early expression of phage genes followed by viral genome replication, late expression of structural genes, virion assembly, and, ultimately, host cell lysis [98]. In contrast, the lysogenic cycle features phage genome integration into the bacterial chromosome, establishing a prophage state that replicates with the host genome. Under specific environmental conditions, the prophage can activate and initiate the lytic cycle, resulting in bacterial lysis [93].

Phage therapy harnesses the bactericidal activity of phages, which occur during the lytic phase of their infectious cycle. Due to the long-standing co-evolution of phages and bacteria, these interactions involve highly specific molecular mechanisms that vary across phage–host systems, making uniform behavior under natural conditions improbable [174]. Therapeutic approaches are broadly categorized as monophage therapy that uses a single phage strain, while polyphage therapy employs carefully designed cocktails of multiple phages to enhance the target spectrum. In clinical practice, both purified lytic phages and optimized phage cocktails can be utilized, enabling personalized treatment strategies based on patient-specific bacterial profiles [176].

Unlike antibiotics, phages exhibit remarkable specificity for bacterial species and strains. This specificity protects commensal gut microbiota, preventing complications such as antibiotic-associated diarrhea, *C. difficile* infections, and comorbidities including diabetes, asthma, and obesity. However, this narrow host range may reduce efficacy in complex infections like burn wounds [177]. While phage cocktails can enhance effectiveness, their development faces technical, and market challenges compared to broad-spectrum antibiotics. Phages activate adaptive immunity, with therapeutic efficacy often linked to phage-mediated bacterial lysis. However, lysis may release endotoxins, causing adverse effects, and phage immunogenicity can lead to neutralizing antibody production, necessitating repeated administrations. Clinical trials suggest low phage toxicity, with immune responses varying by administration route, phage type, and treatment duration [178,179].

Phage cocktails are preferred for their ability to target multiple bacterial receptors simultaneously, reducing resistance development since bacteria would require mutations in multiple receptors to evade infection. Phage therapy has shown promise against drug-resistant *S. aureus* infections, including recurrent prosthetic knee infections (PKIs). Ferry et al. [180] successfully treated three patients with methicillin-sensitive *S. aureus* (MSSA) PKIs using phage cocktails (PP1493, PP1815, and PP1957) combined with antibiotics after failure of DAIR protocol and conventional antibiotic therapy. Intra-articular administration followed by 6–12 weeks of combination antibiotic therapy resulted in significant recovery in two patients, with minimal synovial inflammation, negative C-reactive protein levels, and clinical improvement maintained through the 30-month follow-up [180].

The Endolytix 1 (EC1) cocktail represents an innovative approach against mycobacterial infections, including drug-resistant *M. tuberculosis*. This formulation combines four enzymes (LysA, LysB, isoamylase, and α-amylase) that synergistically degrade the three-layered mycobacterial envelope. LysA, encoded by bacteriophages, plays a crucial role in peptidoglycan layer degradation during bacterial lysis. The remarkable diversity of lysA genes in mycobacteria suggests broad enzymatic activity within this family. LysB, isoamylase, and α-amylase complement LysA activity by targeting other envelope components, enhancing cocktail efficacy. EC1’s design specifically overcomes structural barriers in the mycobacterial envelope for more direct target access [181].

Bacteriophages serve as valuable antimicrobial alternatives in antibiotic-resistant infections, with multiple administration routes including oral, intravenous, intraperitoneal, subcutaneous, intrarectal, topical (particularly for wounds), and nebulized delivery. Combination therapy with antibiotics can produce synergistic effects in the care of wounds resulting from burns, injuries, or surgical procedures; phages show promise in preventing infections at the site of surgery [182].

Phages from *Podoviridae, Siphoviridae*, and *Myoviridae* families are important sources of depolymerases: structural or soluble enzymes that facilitate bacterial host binding and capsule degradation. For instance, *E. coli* K1-specific phage endosialidase E degrades polysialic acid capsules, preventing meningitis and sepsis in animal models. Depolymerases also sensitize multidrug-resistant *A. baumannii* and improve survival in *Pasteurella multocida* infections. The global health challenge of resistant and hypervirulent *K. pneumoniae* clones, associated with liver abscesses, pneumonia, and meningitis, may benefit from such approaches [183].

Growing microbiome research underscores phages’ critical role in maintaining microbial equilibrium. A therapeutics-randomized clinical trial demonstrated *Cutibacterium acnes* phage efficacy in skin microbiota balancing. This phage selectively eliminated pathogenic *C. acnes* strains (RT4, RT5, RT8) associated with acne while preserving beneficial RT6 strains found in healthy skin microbiomes [184].

Nanoparticle delivery systems have emerged as a transformative approach in bacteriophage therapy, significantly enhancing phage stability, target specificity, and biofilm penetration capabilities. Chitosan-based NPs, for instance, provide effective protection against harsh physiological conditions while improving site-specific delivery of therapeutic phages. Concurrently, metallic NPs (gold and silver) have demonstrated remarkable efficacy against resilient biofilms formed by pathogens such as *P. aeruginosa* and *S. aureus* [185].

Recent innovations include advanced nanocomposites like phage-chlorin e6-manganese dioxide (PCM), which combine multiple antimicrobial mechanisms to optimize biofilm penetration. Furthermore, functional modifications such as PEGylation and mannose functionalization have shown promise in enhancing therapeutic precision, enabling targeted delivery to otherwise inaccessible infection sites [186]. These cutting-edge nanoparticle strategies effectively address critical limitations in conventional phage therapy, positioning themselves as powerful tools in the ongoing battle against treatment-resistant infections and persistent biofilm formations. Their continued development represents a crucial frontier in advancing antimicrobial therapeutics, offering novel solutions where traditional approaches have faltered.

## 7. Conclusions

The rise of AMR jeopardizes modern medicine, with drug-resistant infections causing ~5 million annual deaths globally. This review highlights groundbreaking strategies, such as nanotechnology-enhanced drug delivery, engineered antimicrobial peptides, CRISPR-Cas systems, and phage therapy, that redefine the therapeutic arsenal. While preclinical results are promising, clinical adoption faces several hurdles, including cost, regulatory challenges, and scalability issues. A One Health approach, integrating innovative therapies with surveillance and stewardship, is critical to averting a post-antibiotic era. We advise (1) investment in combination therapies, (2) accelerated regulatory pathways for CRISPR-based antimicrobials, and (3) global collaboration to ensure equitable access. Only through multidisciplinary action can we translate these advances into tangible solutions for global health.

## Figures and Tables

**Table 1:** Classes of reported bacterial fatty acid synthesis (FASII) inhibitors.

Inhibitor Class	Target Bacteria	References
Diazaborines	*E. coli*, *S. aureus*	[68]
1,2,3,4-tetrahydro-1H-pyrido [3,4-b]indole derivatives	*S. aureus*, *Enterococcus faecalis*	[69]
1H-imidazole derivatives	*S. aureus*, *E. coli*	[70]
Diphenyl ethers and triclosan analogs	*E. coli*, *M. tuberculosis*	[71]
Naphthyridinones	*S. aureus*, *S. pneumoniae*	[69]
Acrylamide-based compounds	*M. tuberculosis*	[72]
1,5,6,7-tetrahydroindeno [5,6-d]imidazoles	*M. tuberculosis*	[73]
Coumarin derivatives	*M. tuberculosis*, *S. aureus*	[74]
Pyrone/pyridone derivatives	*M. tuberculosis*	[75]
Pyrrolidine-based inhibitors	*P. aeruginosa*	[76]
Quinoline/quinoxaline derivatives	*M. tuberculosis*	[77]
N′-benzoylbenzohydrazides	*M. tuberculosis*	[78]

**Table 2:** Antimicrobial studies using different types of nanocarriers and antibiotics.

Nanoparticles	Drugs	Bacterial Activity	Reference
Gold Nanoparticle	Jellein-I Antimicrobial Peptide	Reduction in biofilm metabolic activity by more than 82%.	[123]
Citric Acid–Magnesium Ferrite Nanocomposite	Amoxicilina	Antimicrobial activity against *S. aureus*, *E. coli,* and *Candida albicans* with 0.312, 0.625, and 1.25 μg/mL MIC, respectively, decreasing biofilm formation against *S. aureus* by 95.34%, *E. coli* by 93.93%, and *C. albicans* by 76.23%.	[124]
Silver Nanoparticles	Ampicillin, Oxacillin, and Penicillin	They showed inhibition of methicillin-resistant *S. aureus* (MRSA) biofilms greater than 90%.	[125]
Selenium Nanoparticles	Levofloxacino	The mature biofilm of *S. aureus* and *E. faecalis* treated with selenium and levofloxacin nanoparticles resulted in a 70% reduction in biofilm biomass compared to the untreated control.	[126]
Silver Nanoflowers	Norfloxacin and Streptomycin	Silver nanoflowers combined with reduced doses of antibiotics such as norfloxacin and streptomycin effectively eliminated resistant E. coli strains. This combination made it possible to reduce the necessary dose of antibiotics by up to 90%.	[127]
Silver Nanoparticles	Gentamicin, Erythromycin, Tetracycline, Ciprofloxacin, Nitrofurantoin, Clindamycin, Trimethoprim-Sulfamethoxazole, Chloramphenicol, Rifampicin, Quinapristin-dAlfopristin, Linezolid, and Cefoxitin	The results showed that the combination of AgNPs with antibiotics increased antibacterial efficacy by up to 182% against *P. aeruginosa* and up to 147% against *S. aureus*. These combinations were also effective in restoring the sensitivity of resistant strains to the antibiotics used.	[128]
Solid Lipid Nanoparticles	Ciprofloxacin, Vancomycin, and Ampicilin	The results showed that the ciprofloxacin-SLN (solid lipid nanoparticle) formulation reduced the burden of *P. aeruginosa* by 64%, while the vancomycin–ampicillin–SLN combination reduced *S. aureus* by 66% at the site of infection compared to untreated groups.	[129]
Polymeric Nanocapsule	Clavanin A	Encapsulation of clavanin A peptide in a polymeric matrix to fight bacterial infections resulted in the inhibition of *S. aureus* growth by 91%, inhibition of *K. pneumoniae* by 20%, and inhibition of *P. aeruginosa* by 39.8%.	[130]
Polymeric Nanoparticle	Ciprofloxacin-Derived Peptide	Nanoparticles of a peptide polymer derived from ciprofloxacin (PAC-NPs), which showed a sterilization rate greater than 91% against Gram-positive and Gram-negative bacteria, minimum inhibitory concentrations (MIC) ranging from 1.0 to 4.0 μg/mL, and low in vitro and in vivo toxicity, with a lower propensity to develop bacterial resistance.	[131]

## Data Availability

Not applicable.
